# Probing the Mechanisms of Inhibitors Binding to Presenilin Homologue Using Molecular Dynamics Simulations

**DOI:** 10.3390/molecules28052076

**Published:** 2023-02-22

**Authors:** Min Wang, Kaifeng Liu, Yingying Ma, Weiwei Han

**Affiliations:** Key Laboratory for Molecular Enzymology and Engineering of Ministry of Education, School of Life Science, Jilin University, 2699 Qianjin Street, Changchun 130012, China

**Keywords:** subunit presenilin 1, inhibitors, Alzheimer’s disease, molecular dynamics simulations, conformational changes

## Abstract

γ-secretase is an intramembrane proteolytic enzyme that is mainly involved in the cleavage and hydrolysis of the amyloid precursor (APP). The catalytic subunit presenilin 1 (PS1) is the catalytic subunit of γ-secretase. Since it was found that PS1 is responsible for Aβ-producing proteolytic activity, which is involved in Alzheimer’s disease, it is believed that reducing the activity of PS1 and preventing or delaying the production of Aβ could help treat Alzheimer’s disease. Consequently, in recent years, researchers have begun investigating the potential clinical efficacy of PS1 inhibitors. Currently, most PS1 inhibitors are only used as a tool to study the structure and function of PS1, and a few inhibitors with a high selectivity have been tested in clinics. Less-selective PS1 inhibitors were found to not only inhibit Aβ production but also inhibit Notch cleavage, which led to serious adverse events. The archaeal presenilin homologue (PSH) is a surrogate protease of presenilin that is useful for agent screening. In this study, we performed 200 ns molecular dynamics simulations (MD) of four systems to explore the conformational changes of different ligands binding to PSH. Our results indicated that the PSH-L679 system formed 3–10 helices in TM4, loosening up TM4 and allowing substrates to enter the catalytic pocket, thereby making it less inhibitory. Additionally, we found that III-31-C can bring TM4 and TM6 closer, resulting in the contraction of the PSH active pocket. Altogether, these results provide the basis for the potential design of newer PS1 inhibitors.

## 1. Introduction

γ-Secretase [1,2,3,4,5], an intramembrane proteolytic enzyme composed of four subunits, is primarily involved in the cleavage and hydrolysis of the amyloid precursor (APP) [6,7] and has been reported to regulate the Notch signaling pathway [8,9]. It has been observed that a decrease in the expression level of any of its subunits can impede the formation of its enzymatic complex.

The four subunits of γ-secretase, PS (presenilin) [1,10,11,12,13,14,15,16], NCT (Nicastrin) [17,18,19,20], APH1(anterior-pharynx-defective-1) [21,22] and PEN2 (Presenilin Enhancer-2) [23,24], are closely and orderly arranged. The functions of the four subunits differ; of these functions, the catalytic subunit of γ-secretase, PS, has substrate-cleaving activity. Dysfunction in the intramembrane protease γ-secretase [1,2,3,4,5] has been associated with Alzheimer’s disease (AD) [1]. Most mutations derived from AD map to the catalytic subunit presenilin 1 (PS1), which is the catalytic subunit of the enzyme [1,4,10,11,12,13,14] and responsible for its Aβ-producing proteolytic activity [1,15,16]. NCT plays a role as a “substrate receptor”. Before the substrate of γ-secretase is cleaved by PS, it is specifically recognized and bound by NCT [17,18,19,20]. APH1 [21,22] and PEN2 [23,24] are two smaller subunits. APH1 is the “scaffold” of the γ-secretase complex assembly and can stabilize active PS. Under necessary conditions, PEN2 contributes to the maturation and cleavage activity of γ-secretase [23,24]. Although it was traditionally believed that the four subunits of γ-secretase could not be separated from each other, recent research showed that only PS1, APH1A and PEN2 cleaved APP and Notch, while the function of NCT was only to stabilize the γ-secretase complex rather than to recognize the substrates [25].

Considering that preventing or delaying the production of Aβ by reducing the activity of γ-secretase could be an effective strategy for treating AD [1], the current focus in this field is research for clinically effective γ-secretase inhibitors. At present, most γ-secretase inhibitors are only used as a tool to study the structure and function of γ-secretase, and only a few have shown promising prospects for potential clinical application. Less-selective γ-secretase inhibitors were shown to not only inhibit Aβ production but also inhibit Notch cleavage, leading to serious toxic side effects [22]. Therefore, it is important to develop new selective γ-secretase inhibitors that can effectively reduce Aβ production without affecting Notch cleavage.

The archaeal presenilin homologue PSH [26] is a protein discovered by Yigong Shi’s team that has a high degree of homology with PS1. This protein can be used as a cost-effective surrogate protease to screen for agents that can regulate the protease activity and the cleavage preference of γ-secretase. Furthermore, this protein can provide valuable insight into the structure and function of γ-secretase, which is essential for understanding the role of this enzyme in the pathogenesis of Alzheimer’s disease.

III-31-C [27] and L682,679 (Abbreviated as L679 below) [28] are aspartyl protease transition-state analogues that mimic gemdiol intermediate and belong to the PS1 mimetic peptide inhibitor. Although they are structurally similar, in vitro assays [26] have demonstrated that L679 is more effective at inhibiting the PS1 homologous alternative PSH than III-31-C. L679 has an IC50 of 0.2 mM compared to an IC50 of 10 μM for III-31-C. However, the mechanisms behind these differences remain largely unknown. Investigating the mechanisms behind these differences could be clinically beneficial as it could provide valuable insight into the structure of PS1 inhibitors.

In this study, we performed 200 ns molecular dynamics simulations (MD) of four systems (PSH, PSH-APP, PSH-L679 and PSH-III-31-C) to investigate the different conformational changes for PSH binding to different ligands (APP and two inhibitors). Our theoretical research might provide useful clues for the design of a new specific inhibitor of PS1. Additionally, this research could help to further our understanding of the structure and function of γ-secretase and its role in the pathogenesis of Alzheimer’s disease.

## 2. Results and Discussion

### 2.1. The Binding Mode of Inhibitors to PSH

**Figure 1:**
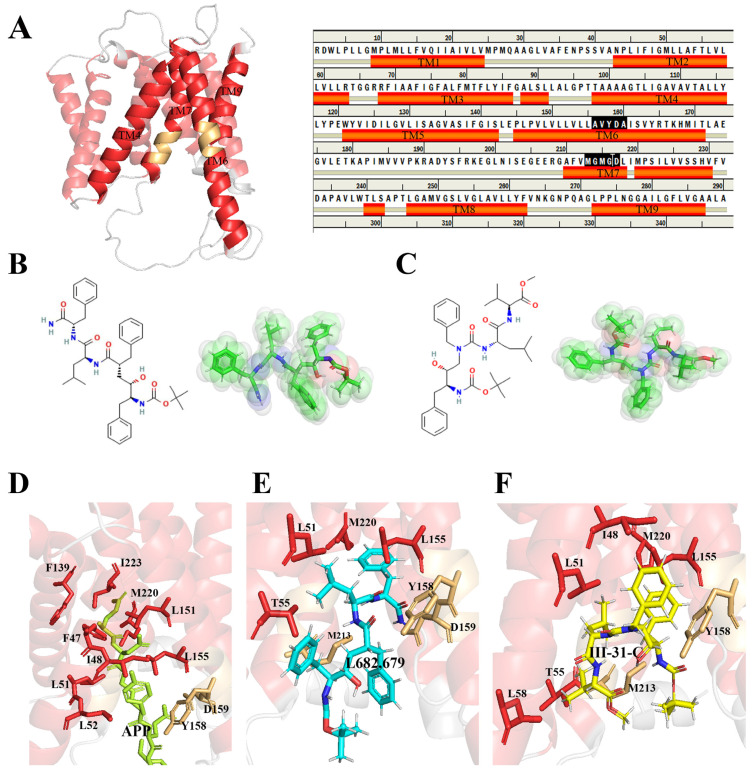
(**A**) Domain composition of PSH (PDB code:4Y6K) and structure sequence (right). The transmembrane helices are shown in red and the catalytic motifs are in a golden color. (**B**) Chemical structure of L679. (**C**) Chemical structure of III-31-C. Binding pocket for (**D**) APP, (**E**) L679 and (**F**) III-31-C. Residues that interacted with the legends are shown as sticks.

The PSH cavity is surrounded by transmembrane helices TM3, TM6, TM7 and TM9, and the motifs (AVYDA on TM6 and MGMGD on TM7) are important for substrate catalyzing. Figure 1A shows the helices in red and the catalytic motifs in a golden color. L679, a peptide-like inhibitor, is located at PSH (PDB code:4Y6K) [29] (Figure 1B,E). APP and the inhibitor, III-31-C (Figure 1C), were docked to PSH with Autodock vina [30] (Figure 1D,F). The docking pose with the lowest energy between the ligands and the PSH was chosen for further study. Figure 1D shows the active residues around APP to PSH. F47, I48, L51, L52, F139, L151, L155, Y158, D159, M220 and I223 were key residues for the PSH binding to APP. Figure 1E shows that L51, T55, L155, Y158, D159, M213 and M220 were key residues for PSH to bind to the inhibitor L679. Figure 1F shows the active residues (I48, L51, T55, l58, L155, Y158, M213 and M220) of PSH around III-31-C. Figure S1A,B displays the interactions between L697 and III-31-C and PSH. Figure S2 shows the interactions between APP and PSH.

### 2.2. Structural Stability and Dynamics Properties of the Four Systems

**Figure 2:**
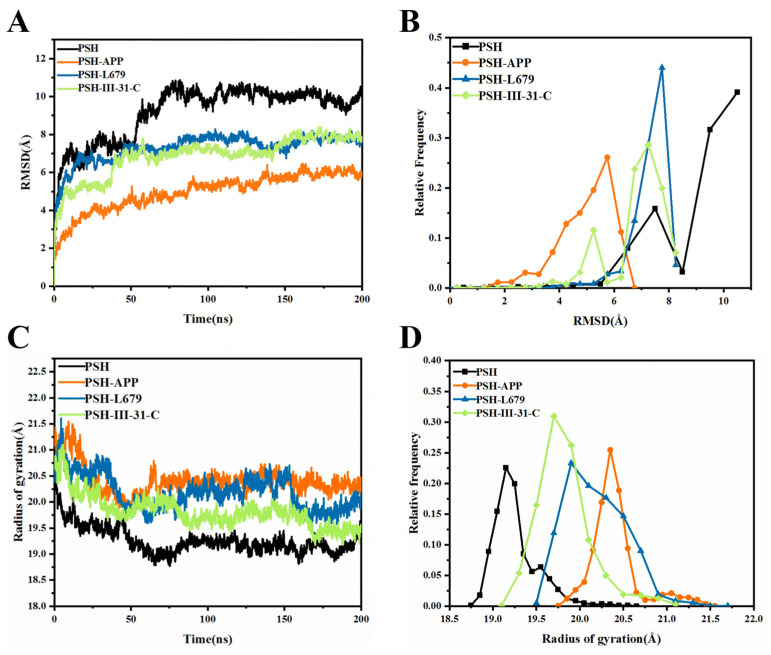
Analysis of structural stability. (**A**) The temporal evolution of the RMSDs from their initial structure of the four systems. (**B**) Relative frequencies of RMSDs for the four systems. (**C**) The radius of gyration over 200 ns MD for the four systems. (**D**) Relative frequencies of radius gyration.

To evaluate the stability of simulations, the root mean square deviation (RMSD) of the CA atoms was calculated (Figure 2A,B and Figure S3A,B). After 75 ns simulations, the RMSD in each MD trajectory reached equilibrium, indicating that all the investigated systems were stable. Moreover, these small mean RMSD values indicated that the investigated four systems had no significant changes. Therefore, the equilibrated 200 ns trajectories can be performed for a post-processing analysis.

The Rg value of the PSH-APP system was higher than the other systems after 50 ns MD simulations, indicating that the PSH-APP system had a larger volume than the other systems (Figure 2C,D and Figure S3C,D). As can be seen from Figure 2C and Figure S3C,D, the Rg values of PSH-III-31-C are significantly less than PSH-L679 after the systems became stable in the three replicas, demonstrating that protein tightness was enhanced by III-31-C binding.

**Figure 3:**
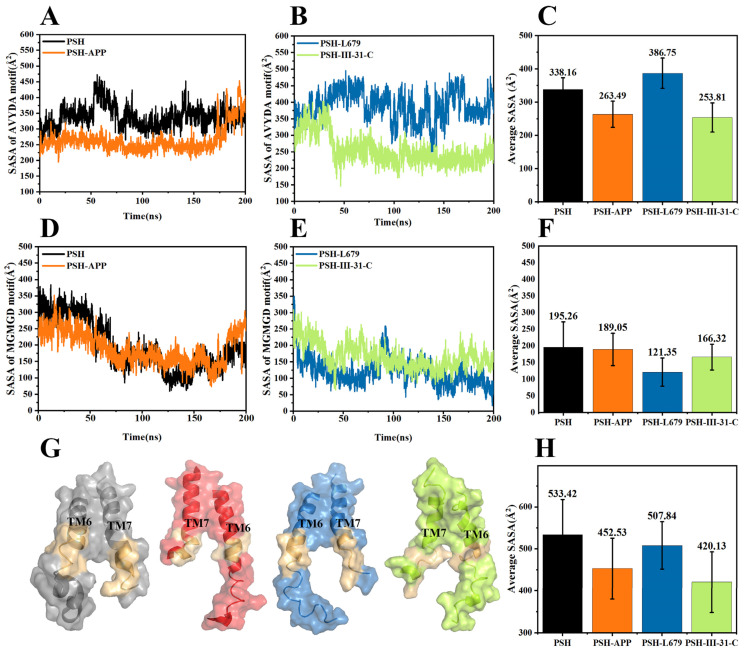
SASA values of the AVYDA catalytic motif of (**A**) PSH and PSH-APP, (**B**) PSH-L679 and PSH-III-31-C during the 200 ns MD. (**C**) Average SASA values of the AVYDA catalytic motif during the 200 ns for the four systems. SASA values of the MGMGD catalytic motif of (**D**) PSH and PSH-APP, (**E**) PSH-L679 and PSH-III-31-C during the 200 ns MD. (**F**) The average SASA values of MGMGD catalytic motif during the 200 ns for the four systems. (**G**) The surface of TM6 and TM7 of the four systems. PSH, PSH-APP, PSH-L679 and PSH-III-31-C are shown from left to right, respectively. The two catalytic motifs are shown in golden color. (**H**) The average sum of the two motifs’ SASA values indicated that the catalytic motifs of PSH-III-31-C had the smallest solvent-accessible surface area.

The solvent-accessible surface area (SASA) was used to predict the number of residues in the outlier regions (surface) of the protein and the number of residues in the hydrophobic core (buried). The SASA values of the AVYDA catalytic motif of PSH and PSH-APP, PSH-L679 and PSH-III-31-C during the 200 ns MD are shown in Figure 3A,B. Figure 3C shows the average SASA values of the AVYDA catalytic motif during the 200 ns MD for the four systems. Figure 3D–F show the SASA values of the MGMGD catalytic motif of PSH and PSH-APP, PSH-L679 and PSH-III-31-C during 200 ns MD (Figure 3E) and the average SASA values of MGMGD catalytic motif during 200 ns for the four systems (Figure 3F). Additionally, Figure 3G illustrates the surface of TM6 and TM7 of the four systems. The average sum of the SASA values of the the two motifs indicates that the catalytic motifs of PSH-III-31-C had the smallest solvent-accessible surface area (Figure 3H). Since the inhibitors were hydrophobic, the smaller SASA value indicated that III-31-C bound more tightly to the active site of PSH.

**Figure 4:**
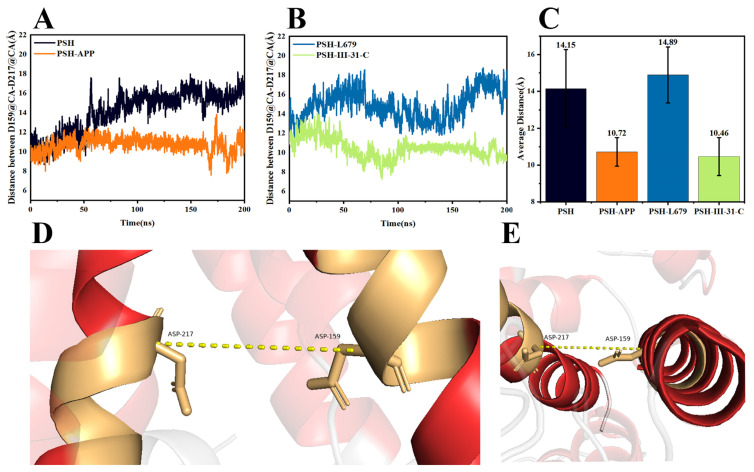
Distance between the Asp159 and Asp217 CA atoms during the 200 ns MD for (**A**) PSH and PSH-APP and (**B**) PSH-L679 and PSH-III-31-C. (**C**) Average distance between the Asp159 and Asp217 of the four systems. (**D**) Schematic diagram of the structure. The dashed line shows the distance between the catalytic residues Asp159 and Asp 217, shown as sticks. (**E**) The distance between catalytic residues from another angle view.

Further, we also explored the distance between the hydrophilic residues Asp159 and Asp217. The distance between the CA atoms of Asp159 on TM6 and Asp217 on TM7 during 200 ns MD is shown in Figure 4 (Figure 4A shows PSH and PSH-APP and Figure 4B shows PSH-L679 and PSH-III-31-C, respectively). The average distance between the active residues, Asp159 and Asp217, of the four systems is shown in Figure 4C. Figure 4D,E illustrate the distance between the catalytic residues Asp159 and Asp 160. Based on the observations, the distance in PSH-III-31-C was the closest. A closer distance between the two active residues indicates that they passed through a narrower channel, making it difficult for the substrate to enter. Our results were consistent with the experimental results that III-31-C is a better inhibitor for PSH than L679 [2]. Altogether, the III-31-C binding brought TM6 and TM7 closer together, resulting in the PSH active site contract and thereby aiding the binding.

### 2.3. Comparison of the Conformational Changes of the Four Systems

**Figure 5:**
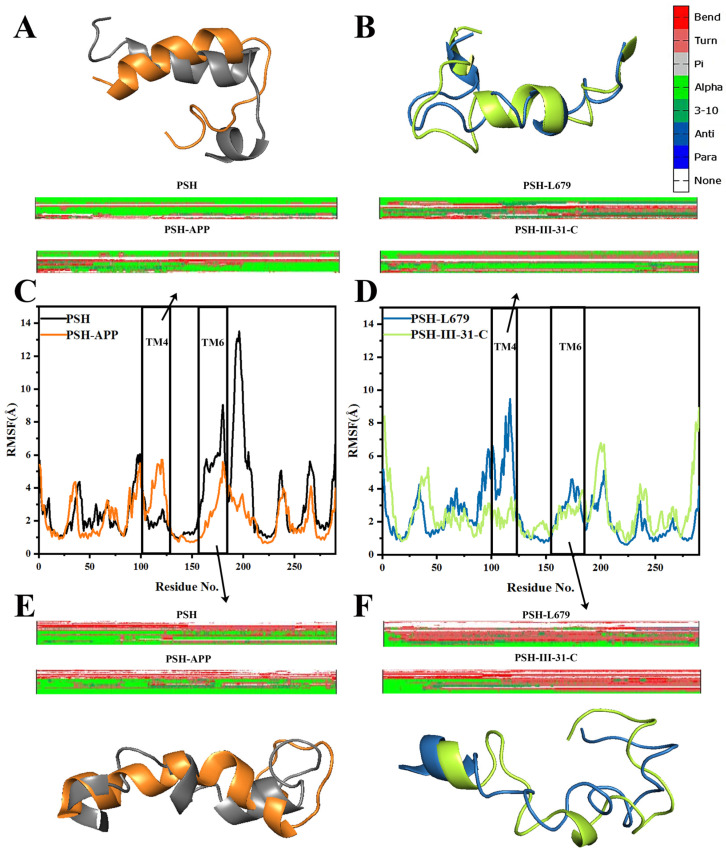
DSSP and structures comparison of TM4, (**A**) PSH and PSH-APP and (**B**) PSH-L679 and PSH-III-31-C. The RMSFs of CA atoms in the four systems. (**C**) PSH compared with PSH-APP. (**D**) PSH-L679 compared with PSH-III-31C. The TM4 and TM6 regions are shown as rectangles. DSSP and structures comparison of TM6, (**E**) PSH and PSH-APP and (**F**) PSH-L679 and PSH-III-31-C.

Here, we analyzed the RMSF plot of the four systems (Figure 5C,D and Figure S3E,F) and the secondary structures of residues 100–125 at TM4 and residues 155–180 at TM6 (Figure 5A,B,E,F). In all three replicates, PSH-III-31-C exhibited lower a RMSF than PSH-L679 in both TM4 and TM6 regions, indicating better rigidty.

Compared to the PSH-APP system, TM6 was affected by the hydrophobic interaction of the two inhibitors, with fewer helices and more turns, in both the PSH-L679 and PSH-III-31-C systems. The averaged probabilities of the α-helix and turn over three replicates for PSH-APP, PSH-L679 and PSH-III-31-C of residues 158–171 on TM6 are shown in Table S1, indicating the good repeatability of secondary structure changes. This may be a similarity in the mechanism of PSH inhibition between the two inhibitors (Figure 5E,F).

From Figure 5B, it can be seen that the PSH-L679 system formed 3–10 helices in TM4, loosening up the helix and facilitating the substrate entering the catalytic pocket, reducing its inhibition ability. This could be attributed to the large steric hindrance of L679. In contrast, the TM4 of III-31-C contained an alpha helix that maintained rigidity to prevent the substrate from entering the active pocket. This reliability can be demonstrated by the averaged probabilities of the α-helix and 3–10 helix for TM4 in PSH-L679 and PSH-III-31-C over three replicates (see Table S2). This may be one of the reasons for the difference in the inhibitory ability of the two inhibitors.

### 2.4. Protein Network Analysis

**Figure 6:**
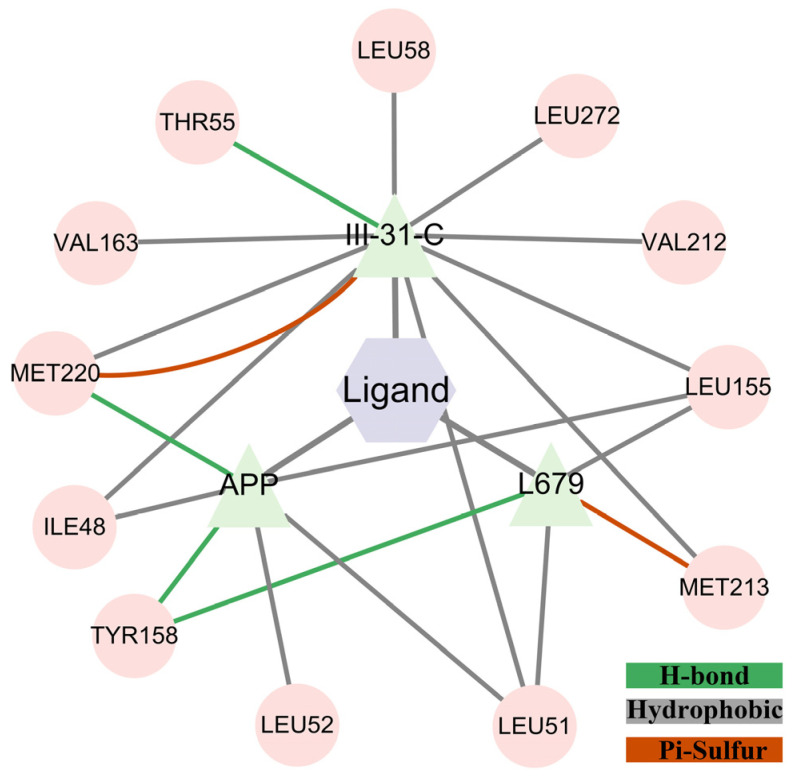
Interaction networks analysis between ligands and PSH.

Figure 6 shows the interaction network analysis between ligands and PSH. The nodes represent residues, and the edges represent interactions. The gray color indicates the hydrophobic forces, green represents the hydrogen bonds and red represents pi–sulfur. Figure 6 shows that more residues interacted with III-31-C than L679. In addition, the III-31-C inhibitor and Leu272, located at TM9, had hydrophobic interactions that were useful for III-31-C binding to PSH.

### 2.5. Quantum Chemical Calculation

**Figure 7:**
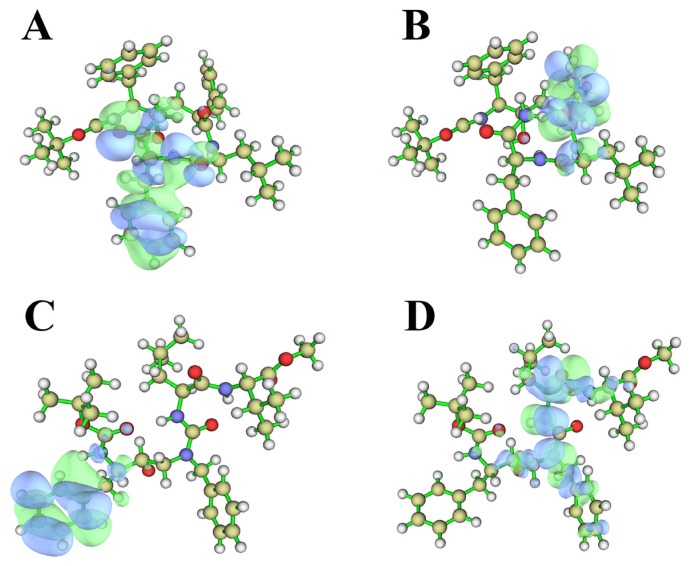
(**A**) Molecular structure and LUMO orbitals of L679. (**B**) Molecular structure and HOMO orbitals of L679. (**C**) Molecular structure and LUMO orbitals of III-31-C. (**D**) Molecular structure and HOMO orbitals of III-31-C.

To explore the mechanism of the PSH inhibitors, quantum chemical calculations were conducted for the two compounds. The HOMO-LUMO energy gap represents the energy difference between the HOMO orbit and the LUMO orbit (Figure 7A–D), which depend on all coordinates of the system, providing a more efficient sampling method than a geometrical reaction coordinate to better reflect the activities of the compounds. The HOMO-LUMO energy gap of III-31-C was 516.50 KJ/mol, and the HOMO-LUMO energy gap of L679 was 558.70 KJ/mol. Considering that a lower energy can result in electronic transitions and the easy formation of new interactions, III-31-C is capable of forming more interactions than L679, including hydrogen bonds (H-bonds) and van der Waals (vdW) interactions. As a result, III-31-C binds to PSH more effectively than L679.

### 2.6. Dynamical Cross-Correlation Matrix and Principle Component Analysis

**Figure 8:**
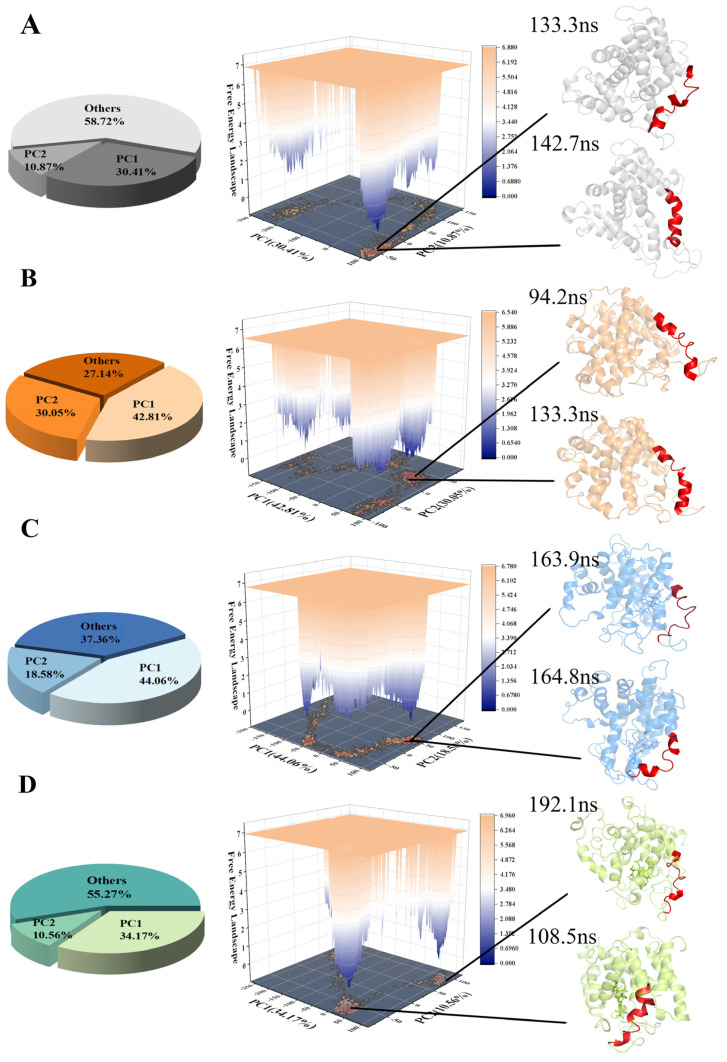
The free energy landscape for the following three systems: (**A**) PSH, (**B**) PSH-APP, (**C**) PSH-L679 and (**D**) PSH-III-31C. Representative conformations of the low-energy regions are displayed, and residues A156–A173 are shown in red.

A PCA analysis of the CA atoms, performed for the four systems, is shown in Figure 8A–D). PC1 and PC2 accounted for more than 40%, reflecting the reliability of the results. The red region shows the conformational changes in TM6. The stable conformations are consistent with the previous analysis.

**Figure 9:**
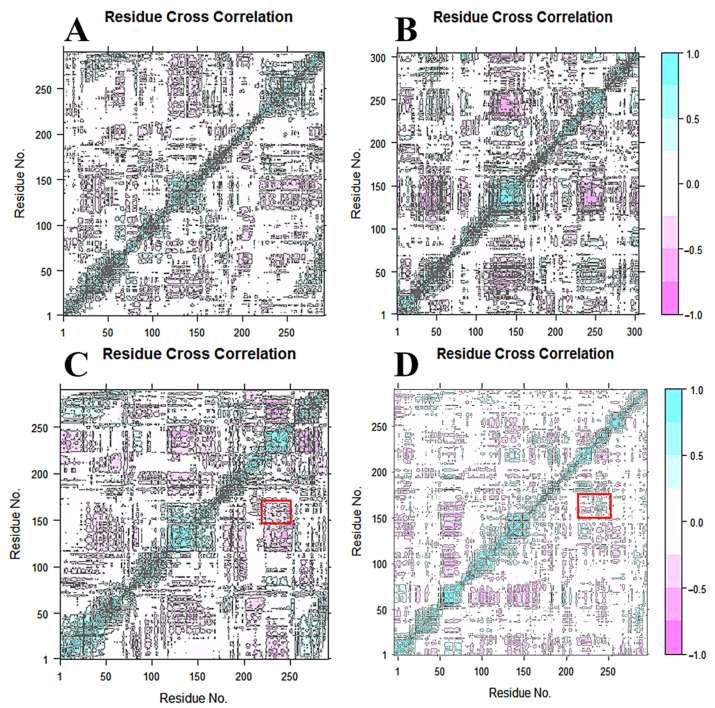
The dynamic cross-correlation map for the 200 ns MD simulation trajectories of the four systems: (**A**) PSH, (**B**) PSH-APP, (**C**) PSH-L679 and (**D**) PSH-III-31-C. The positive regions are shown in cyan and the negative regions are colored in pink, representing correlated and anti-correlated motions between residue CA atoms. Red rectangles show the action between TM6 and TM7.

The dynamic cross-correlation map for the 200 ns MD simulation trajectories of the four systems is shown in Figure 9A–D, respectively. The positive regions are shown in cyan and the negative regions are shown in pink, representing the correlated and anti-correlated motions between residue CA atoms. Red rectangles show the action between TM6 and TM7. From Figure 7C, it can be observed that the pink areas show the backward direction movement of TM6 and TM7 in PSH-L679, while in Figure 7D, the cyan areas show the opposite direction movement in PSH-III-31-C. This is consistent with the previous results, which showed that the distance between ASP159 and ASP217 was decreased in PSH-L679 and increased in PSH-III-31-C. Once again, the binding of III-31-C was proven to constrict the PSH active site; hence, its inhibitory ability was greater than that of L679.

### 2.7. MM-PBSA Study

The results of MM-PBSA are shown in Table 1. The binding free energy of PSH-APP was −48.32 ± 1.35 KJ/mol, while the binding free energy of PSH-L679 was −31.78 ± 0.54 KJ/mol. This was even less than the binding free energy of the APP, indicating that the inhibitory effect was highly concentration-dependent. The binding free energy of PSH-III-31-C was −65.02 ± 0.35 KJ/mol: the strongest binding force. Overall, the results were consistent with the experiment [2], with III-31-C demonstrating a better binding affinity to PSH than L679.

**Figure 10:**
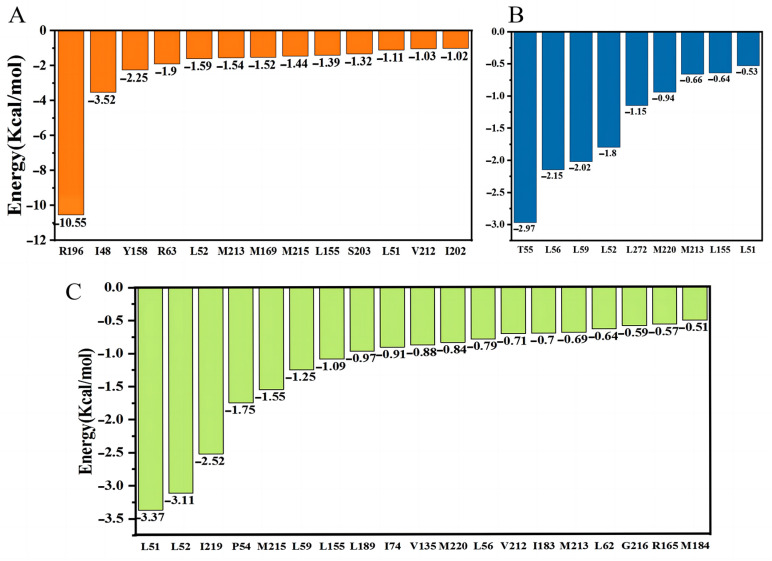
The binding energy contribution of residues (**A**) PSH-APP, (**B**) PSH-L679 and (**C**) PSH-III-31-C.

Figure 10A–C show the binding energy contribution of residues in three PSH–ligand systems. From Figure 10A, R196, I48, R63, L52, M213, M169, L165, S203, L51, V212 and I202 were key residues for APP binding to PSH. T55, L56, L59, L52, L272, M220, M213, L155 and L51 were key residues for L679 binding to PSH (Figure 10B). Comparatively, L56, L52, I219, P54, M215, L59, L155, L189, I74, V135, M220, L56, V212, I183, M213, L62, G216, R165 and M184 were key residues for III-31-C binding to PSH (Figure 10C). Altogether, we found that more residues were involved in III-31-C binding to PSH, which decreased the free energy between III-31-C and PSH.

## 3. Materials and Methods

### 3.1. System Preparation

The 3D structures of PSH-L679 (PDB code: 4Y6K) were obtained from the protein database (www.rcsb.org (accessed on 21 April 2022)) [29]. Using the UCSF Chimera software, PSH was prepared by removing the ligand, repeated monomers and water, and the Modeller plugin was used to model all the missing structures. The structure of APP was then separated from PSH-APP (PDB code: 3SV1) [31]. The III-31-C compound was modeled using Discovery Studio [32]. Next, Gaussian 09 [33] was used to optimize the structure at the level of B3LYP/6-31G* to obtain the optimal conformation for molecular docking. APP and III-31-C were docked to the active site of PSH with Autodock Vina [30] to form the PSH-APP and PSH-III-31-C systems, respectively. The size of the docking box was set to x = 50, y = 40 and z = 50, and the spacing between grid points was set to 0.375 Å. The lowest energy structures were selected from docking results as the initial structures for the MD simulations. Lastly, the four systems, including the free PSH, PSH-APP, PSH-L679 and PSH-III-31-C, were constructed.

### 3.2. Molecular Dynamics Simulations

Molecular dynamics (MD) simulations were performed using the PMEMD engine provided with the AMBER 16 [34] package, in which the FF99SB AMBER force field [35] was used to describe the systems. The TIP3P water model [36] was used, and edge effects were prevented using periodic boundary conditions (PBCs) during the simulation time. The distance between the solute surface and the box was set to 12 Å. Then, appropriate amounts of Na+ were added to the system to neutralize the system. All bonds involving hydrogen atoms were constrained using the SHAKE algorithm [37]. The particle mesh Ewald (PME) algorithm [38] was used to handle non-bonded electrostatic interactions. An initial minimization of 3000 steps was conducted with an applied restraint potential of 10 kcal/mol for the enzyme. An additional full minimization of 1000 steps was further carried out using a conjugate gradient algorithm without restraints. A gradual heating MD simulation from 0 K to 300 K was executed for 50 ps for the systems to maintain a fixed number of atoms and volume. Solutes within the systems were imposed with a potential harmonic restraint of 10 kcal/mol and a collision frequency of 1 ps. Following heating, an equilibration estimating 500 ps of each system was conducted with a constant operating temperature of 300 K. The system’s pressure was maintained at 1 bar using the Berendsen barostat [39], and the total simulation trajectory was 200 ns. The simulations coincided with the isobaric–isothermal ensemble (NPT), with randomized seeding, a constant pressure of 1 bar (maintained by the Berendsen barostat), a pressure-coupling constant of 2 ps, a temperature of 300 K, and a Langevin thermostat [40] with a collision frequency of 1 ps. Each MD simulation was performed three times (See Figure S3).

### 3.3. Trajectory Analysis

A trajectory analysis, including the RMSD, Rg, SASA, RMSF and a dictionary of secondary structures, was computed using Amber16’s Cpptraj module [41]. Representative structures were obtained using K-means clustering.

### 3.4. Protein Structure Network Analysis

The network analysis of the proteins to different ligands was analyzed using generated graphs, in which the average structures were used and each residue was defined as a node. They were connected by edges corresponding to non-covalent interactions. The Cytoscape [42] software was used for graphing.

### 3.5. Quantum Chemical Calculation

Quantum chemical calculations of L679 and III-31-C were performed using the B3LYP function at the 6-31G* set in the Gaussian 09 [33] software. Multiwfn [43] was used to calculate the HOMO-LUMO energy gap and plot.

### 3.6. MM-PBSA Calculations

The accurate calculation of protein–protein binding free energy is of great importance in biological and medical science. This work used the molecular mechanics/Poisson–Boltzmann surface area (MM/PBSA) method to explore the ligands’ binding affinity to PSH [44,45].

The binding free energy (ΔG_bind_) can be expressed by Equation (1).

ΔG_bind_ = ΔH − TΔS (1)

The changes in the protein and ligand upon binding were similar in all systems, with very small entropy differences; therefore, the calculation of the solvate entropy term is omitted. The enthalpy change (ΔH) was computed as the sum of changes of the gas phase energy (ΔE_MM_), and the solvation-free energy (ΔG_sol_), averaged over a conformational ensemble generated by MD simulations (Equation (2)):

ΔH = ΔE_MM_ + ΔG_sol_ (2)

ΔE_MM_ was estimated using the following formula:

ΔE_MM_ = ΔE_ele_ + ΔE_vdW_ + ΔE_int_ (3)

where ΔE_ele_, ΔE_vdW_ and ΔE_int_ represented the electrostatic, vdW energies and internal energies corresponding to the bond, angle and dihedral energies, respectively.

In this study, the conformational structures of the protein–ligand complex, protein and ligand were obtained from a single MD trajectory (only complex trajectory) that regarded the protein–ligand structure as a rigid body. Thus, the ΔE_int_ between the complex and the isolated components could offset each other because this energy term was calculated from the same MD simulated trajectory.

Further, only the ΔE_ele_ and ΔE_vdW_ of Equation (3) were studied in the following work.

ΔG_sol_ was used to indicate the sum of the polar solvation-free energy (ΔG_pb_) and non-polar solvation-free energy(ΔG_np_).

ΔG_sol_ = ΔG_pb_ + ΔG_np_ (4)

ΔG_pb_ was determined by solving the linearized Poisson–Boltzmann equation using the PBSA program in the AMBER 16 suite [34]. Then, 50 snapshots were extracted from the final trajectory for MM/PBSA calculation.

## 4. Conclusions

In this study, we used a 200 ns molecular dynamics simulation to determine the mechanisms responsible for the differences in the inhibitory effects in four systems (PSH, PSH-APP, PSH-L679 and PSH-III-31-C). The conformational change showed that during the 200 ns MD simulation, both the inhibitors made TM6 rigid to prevent substrate entry, whereas the excessive spatial site resistance of L679 elevated the flexibility of TM4, preventing it from binding tightly to the PSH. In addition, III-31-C binding caused a contraction of the PSH active site, allowing III-31-C to bind tightly to PSH and inhibit it better. Quantum chemical calculations also showed that III-31-C is more likely to interact with PSH. Lastly, when compared to L679, the binding free energy of III-31-C was lower, once again indicating a better binding capacity. These results revealed the mechanism responsible for the difference in the effect of the two inhibitors, III-31-C and L679, and could be used as a referential basis for designing new inhibitors to treat Alzheimer’s disease.

## Figures and Tables

**Table 1:** The results of MM-PBSA(KJ/mol).

System	PSH-APP	PSH-L679	PSH-III-31-C
ΔE_vdW_	−92.80 ± 2.11	−70.08 ± 0.42	−90.25 ± 0.43
ΔE_ele_	−177.48 ± 4.53	−30.80 ± 0.76	−20.07 ± 0.40
ΔG_solv_	221.95 ± 5.44	69.09 ± 0.78	45.31 ± 0.64
ΔG_gas_	−270.27 ± 6.47	−100.88 ± 0.90	−110.33 ± 0.68
ΔG_total_	−48.32 ± 1.35	−31.78 ± 0.54	−65.02 ± 0.35

## Data Availability

Not applicable.

## Supplementary Materials

The following supporting information can be downloaded at: https://www.mdpi.com/article/10.3390/molecules28052076/s1, Figure S1: (A) The interactions between L697 and PSH. (B) The interactions between III-31-C and PSH. Figure S2: The interactions between APP and PSH. Figure S3: RMSD, Rg and RMSF values of replicas. Table S1: α-helix and Turn probabilities of residues 158–171 on TM6 for PSH-APP, PSH-L679 and PSH-III-31-C. Table S2: α-helix and 3–10 helix probabilities of residues 104-113 on TM4 for PSH-L679 and PSH-III-31-C.

## Sample Availability

Samples of the compounds are not available from the authors.
