# Supplementary material for: Probing the Mechanisms of Inhibitors Binding to Presenilin Homologue Using Molecular Dynamics Simulations
Source: Molecules. 2023 Feb 22;28(5):2076. doi: 10.3390/molecules28052076 (PMC10004098; doi:10.3390/molecules28052076)
Supplement: Supplementary file 1 [file molecules-28-02076-s001.zip › molecules-2172331-supplementary.pdf]

Figure S1. (A) The interactions between L697 and PSH. (B) The interactions between III-31-C and PSH.

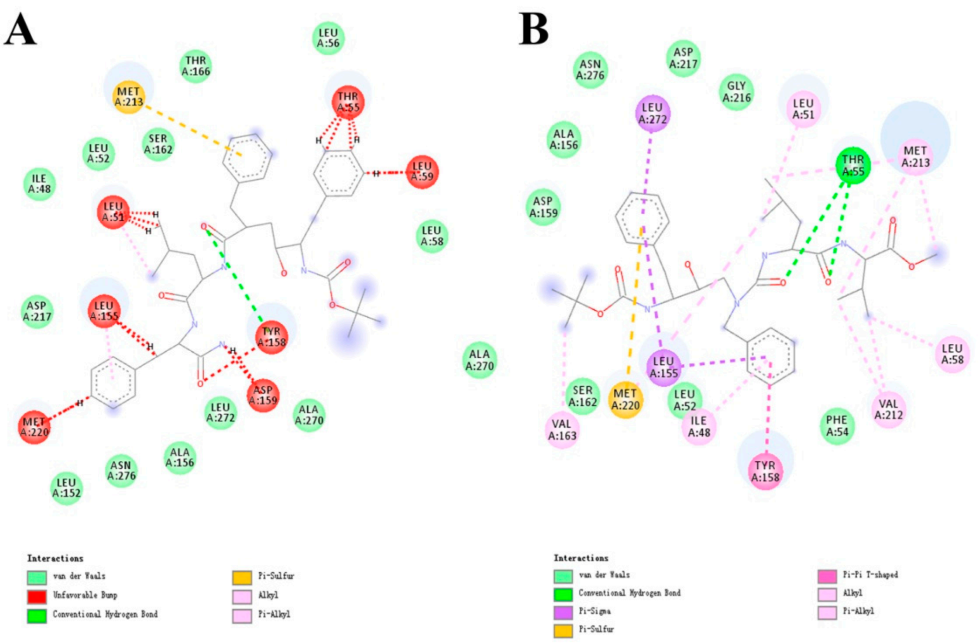

Figure S2. The interactions between APP and PSH.

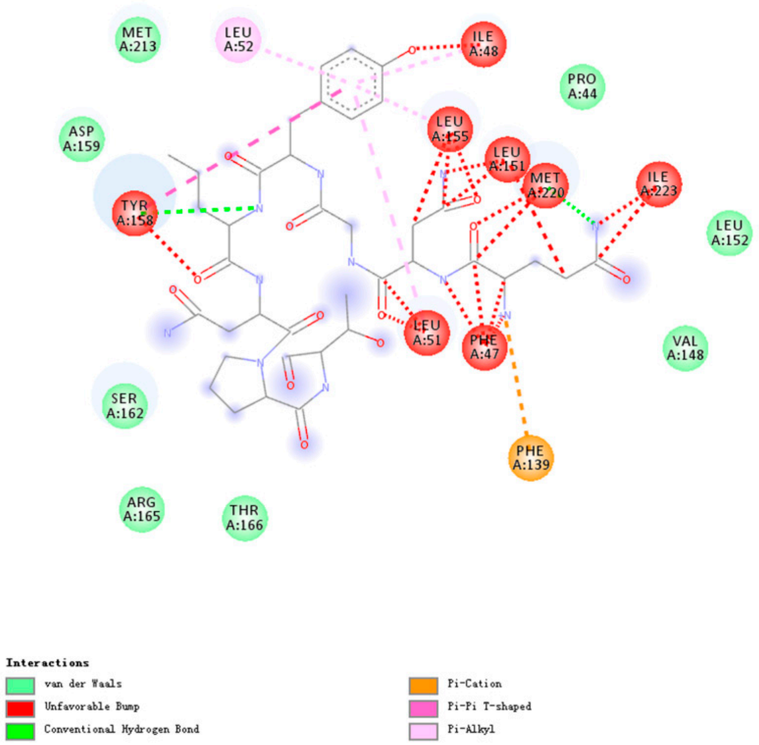

Figure S3. RMSD(**A**,**B**), Rg (**C**,**D**) and RMSF(**E**,**F**) values of replicates.

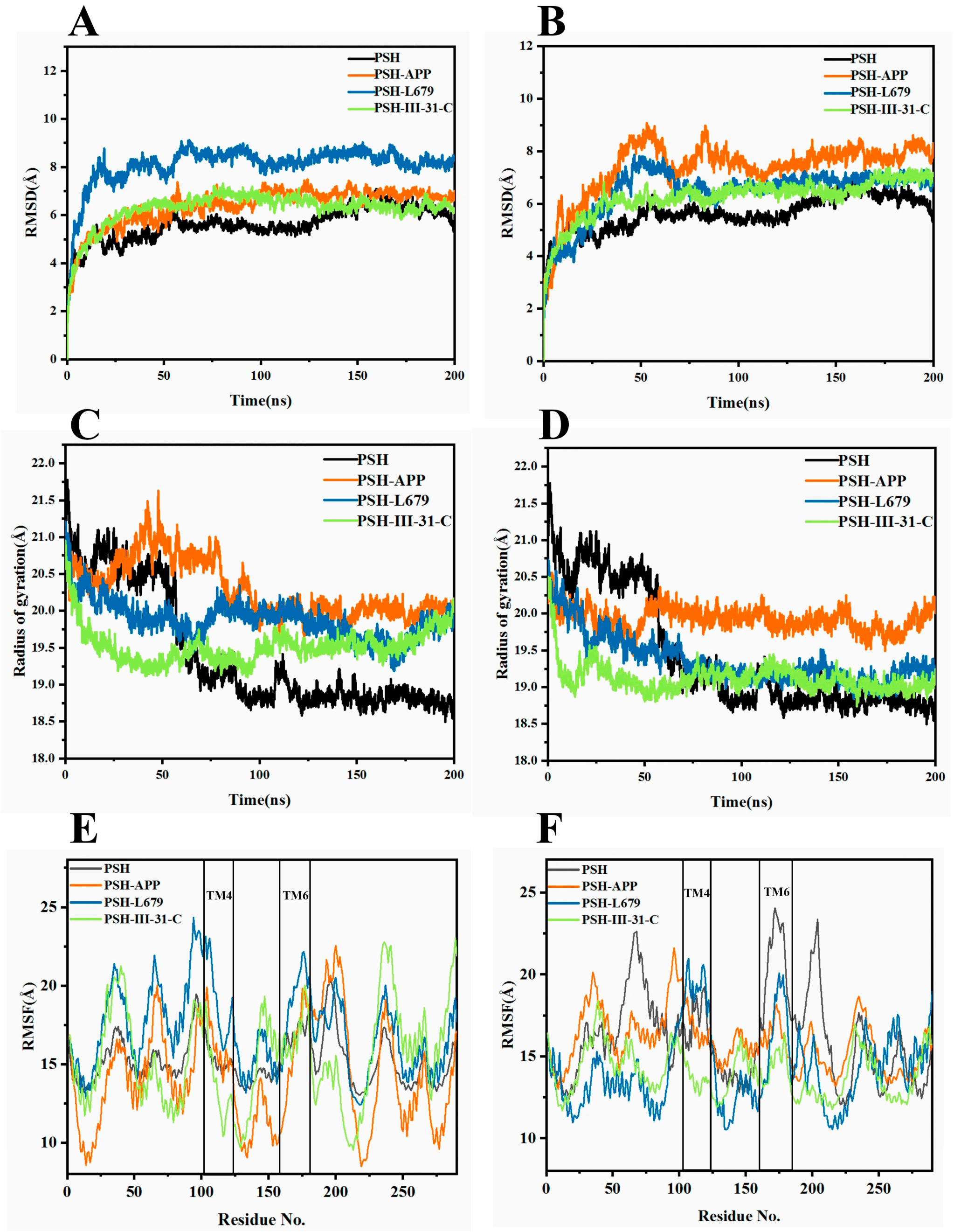

Table S1. α-helix and Turn probabilities of residues 158-171 on TM6 for PSH-APP, PSH-L679 and PSH-III-31-C.

Residues	PSH-APP		PSH-L679		PSH-III-31-C	
	Alpha	Turn	Alpha	Turn	Alpha	Turn
158	0.91±0.08	0.04±0.02	0.57±0.23	0.13±0.12	0.58±0.05	0.40±0.08
159	0.89±0.05	0.04±0.03	0.33±0.07	0.14±0.06	0.14±0.09	0.84±0.06
160	0.39±0.13	0.26±0.15	0.18±0.08	0.30±0.17	0.12±0.05	0.40±0.13
161	0.17±0.04	0.62±0.07	0.11±0.07	0.43±0.27	0.12±0.06	0.38±0.11
162	0.38±0.16	0.21±0.08	0.10±0.03	0.41±0.07	0.15±0.07	0.06±0.03
163	0.37±0.04	0.23±0.14	0.02±0.02	0.44±0.18	0.12±0.11	0.07±0.05
164	0.37±0.05	0.19±0.06	0.02±0.01	0.95±0.05	0.18±0.09	0.52±0.15
165	0.34±0.19	0.20±0.11	0.04±0.01	0.95±0.05	0.17±0.09	0.52±0.06
166	0.25±0.18	0.19±0.04	0.03±0.03	0.92±0.12	0.19±0.15	0.79±0.15
167	0.42±0.11	0.46±0.09	0.12±0.01	0.70±0.14	0.23±0.12	0.69±0.14
168	0.41±0.15	0.48±0.18	0.13±0.06	0.68±0.08	0.14±0.05	0.77±0.09
169	0.55±0.09	0.34±0.07	0.14±0.06	0.54±0.18	0.15±0.04	0.73±0.06
170	0.67±0.11	0.23±0.08	0.12±0.09	0.61±0.09	0.37±0.13	0.36±0.05
171	0.72±0.15	0.16±0.05	0.07±0.06	0.66±0.13	0.35±0.02	0.40±0.09

Table S2. α-helix and 3-10 helix probabilities of residues 104-113 on TM4 for PSH-L679 and PSH-III-31-C.

Residues	PSH-L679		PSH-III-31-C	
	3-10	Alpha	3-10	Alpha
104	0.40±0.04	0.03±0.01	0.03±0.01	0.06±0.03
105	0.41±0.11	0.02±0.01	0.07±0.05	0.53±0.16
106	0.45±0.15	0.04±0.02	0.04±0.03	0.43±0.08
107	0.49±0.12	0.12±0.07	0.08±0.02	0.78±0.16
108	0.56±0.04	0.11±0.07	0.02±0.01	0.77±0.09
109	0.58±0.08	0.13±0.09	0.04±0.03	0.62±0.18
110	0.52±0.09	0.12±0.02	0.06±0.05	0.74±0.09
111	0.18±0.06	0.04±0.03	0.10±0.01	0.69±0.13
112	0.34±0.17	0.15±0.06	0.11±0.04	0.79±0.07
113	0.36±0.09	0.18±0.06	0.08±0.04	0.78±0.09
